# Supplementary figures and images for: Two unequally redundant "helper" immune receptor families mediate Arabidopsis thaliana intracellular "sensor" immune receptor functions
Source: PLoS Biol. 2020 Sep 14;18(9):e3000783. doi: 10.1371/journal.pbio.3000783 (PMC7514072; doi:10.1371/journal.pbio.3000783)

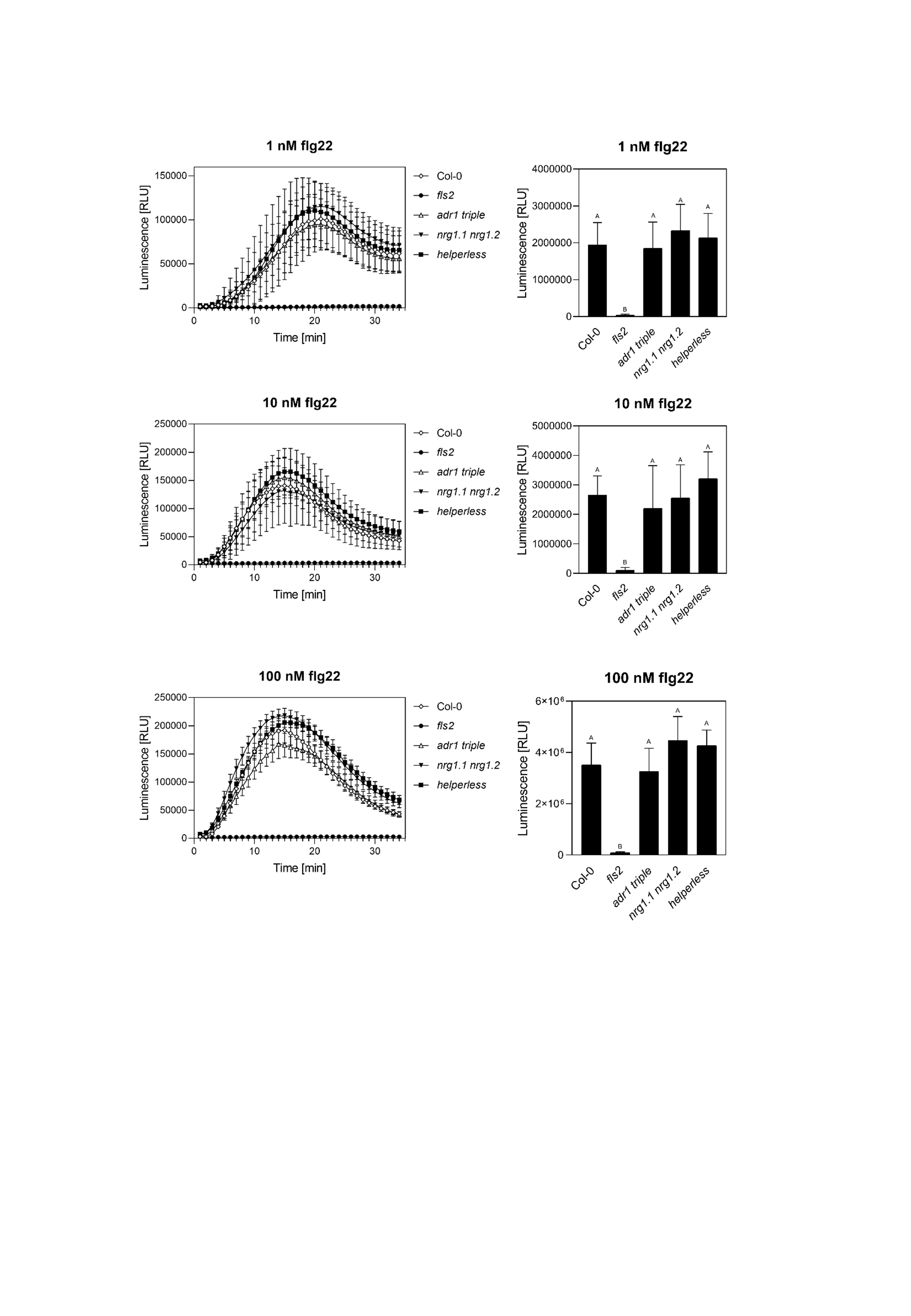

Supplement: S1 Fig — Left panels: Oxidative burst in leaves of the indicated genotypes after addition of 1 nM, 10 nM, or 100 nM flg22. Results are means × SD (n = 6). Right panels: Total ROS production over 34 minutes after flg22 treatment. Values are means × SD (n = 6). Letters indicate statistically significant differences following ANOVA with Tukey’s test (α = 0.05). Experiment was done 3 times. Underlying numerical data are provided in S1 Data. (TIF) [file pbio.3000783.s001.TIF]

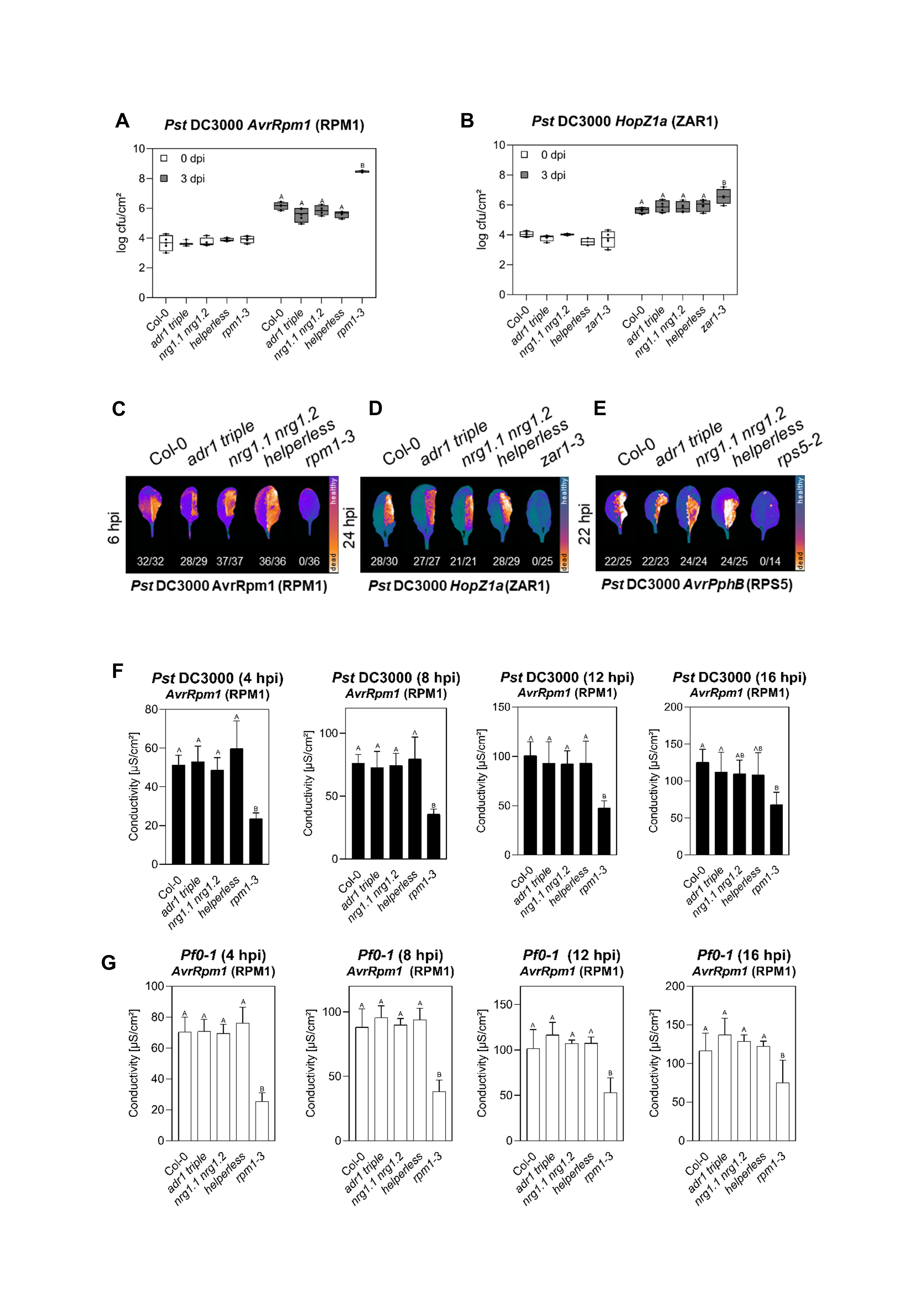

Supplement: S2 Fig — (A, B) Six-week-old plants were hand-infiltrated with Pst DC3000 (A), AvrRpm1 (OD600 = 0.001), or (B) HopZ1a (OD600 = 0.001), and bacterial growth was assessed at 0 and 3 dpi. Box limit represents upper and lower quartile; maximum and minimum values are displayed in whiskers. The middle line shows the median, the cross the mean cfu/cm2. Dots represent 4 technical replicates (leaf discs) in one experiment (biological replicate). Experiment was done 3 times with similar results. Letters indicate statistically significant differences following ANOVA with Tukey’s test (α = 0.05). (C–E) The right leaf half of 6-week-old plants was hand-infiltrated with (C) Pst DC3000 AvrRpm1 (OD600 = 0.1), (D) Pst DC3000 HopZ1a (OD600 = 0.1) or (E) Pst DC3000 AvrPphB (OD600 = 0.1). A Typhoon laser scanner was used to detect autofluorescence of dead leaf tissue at indicated time points. Representative leaves shown in a false color scale (black to blue: healthy leaf tissue, orange to white: dead). NLRs activated in infection experiments shown in A–E are indicated in parenthesis. (F, G) Leaves of 6-week-old A. thaliana plants were fully hand-infiltrated with either (F) Pst DC3000 expressing AvrRpm1 (OD600 = 0.1) or (G) Pf0-1 expressing AvrRpm1 (OD600 = 0.2). Twenty-five leaf discs were collected and rinsed in deionized water, and conductivity of 5 leaf discs immersed in 3 mL deionized water was measured at 4, 8, 12, and 16 hpi. Values are means of conductivity [μS/cm2] (n = 5). Letters indicate statistically significant differences following ANOVA with Tukey’s test (α = 0.05). Experiment was done twice with similar results. Underlying numerical data are provided in S1 Data. (TIF) [file pbio.3000783.s002.TIF]

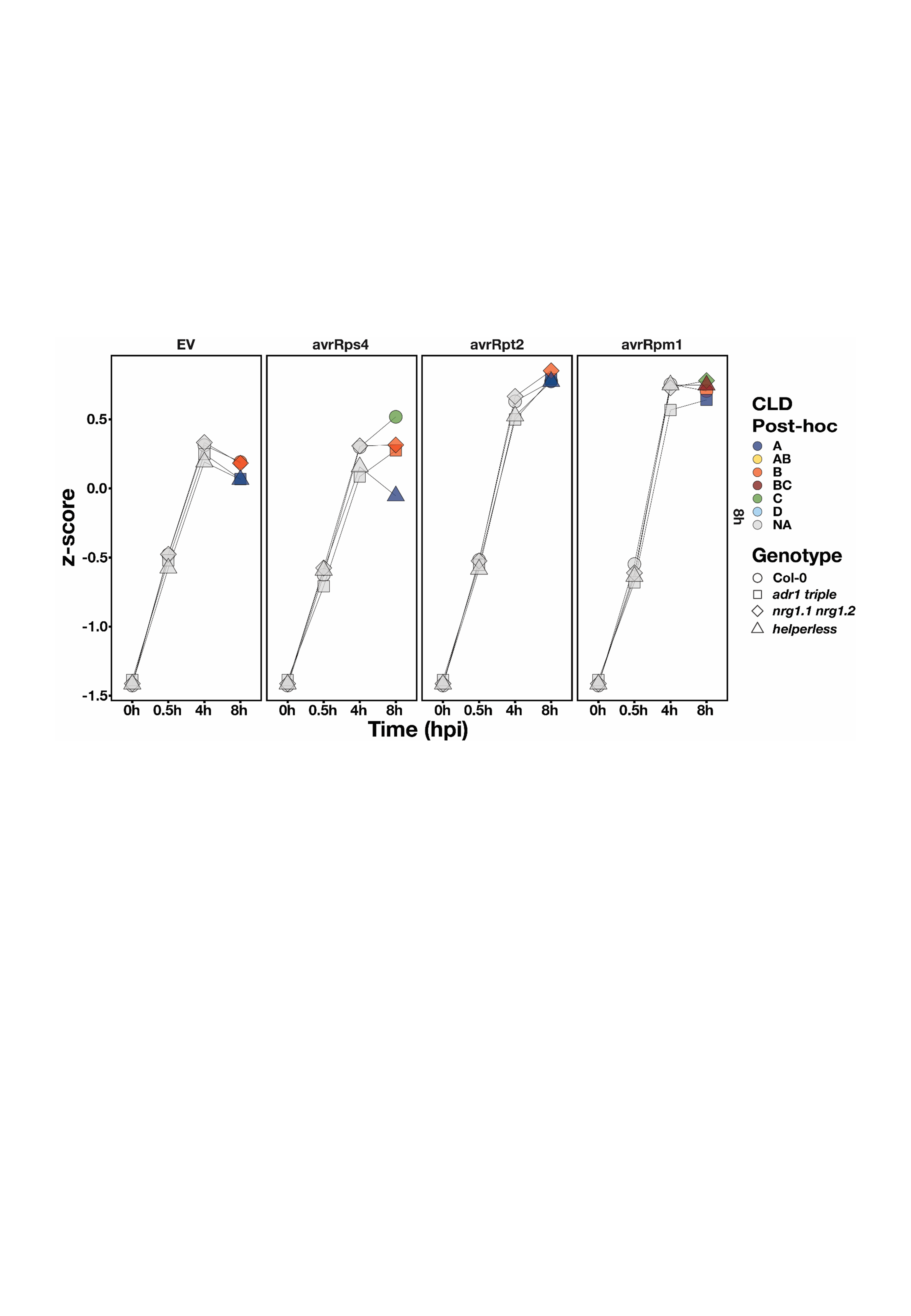

Supplement: S3 Fig — Expression profile of PTI-regulated genes at 0.5 hpi, 4 hpi, and 8 hpi with Pf0-1 EV, Pf0-1 AvrRps4, Pf0-1 AvrRpt2, or Pf0-1 AvrRpm1. PTI genes, which are induced by Pf0-1 EV infections, are over-induced during RPS4 ETI in an RNL-dependent manner. Underlying numerical data are provided in S1 Data. (TIF) [file pbio.3000783.s003.TIF]

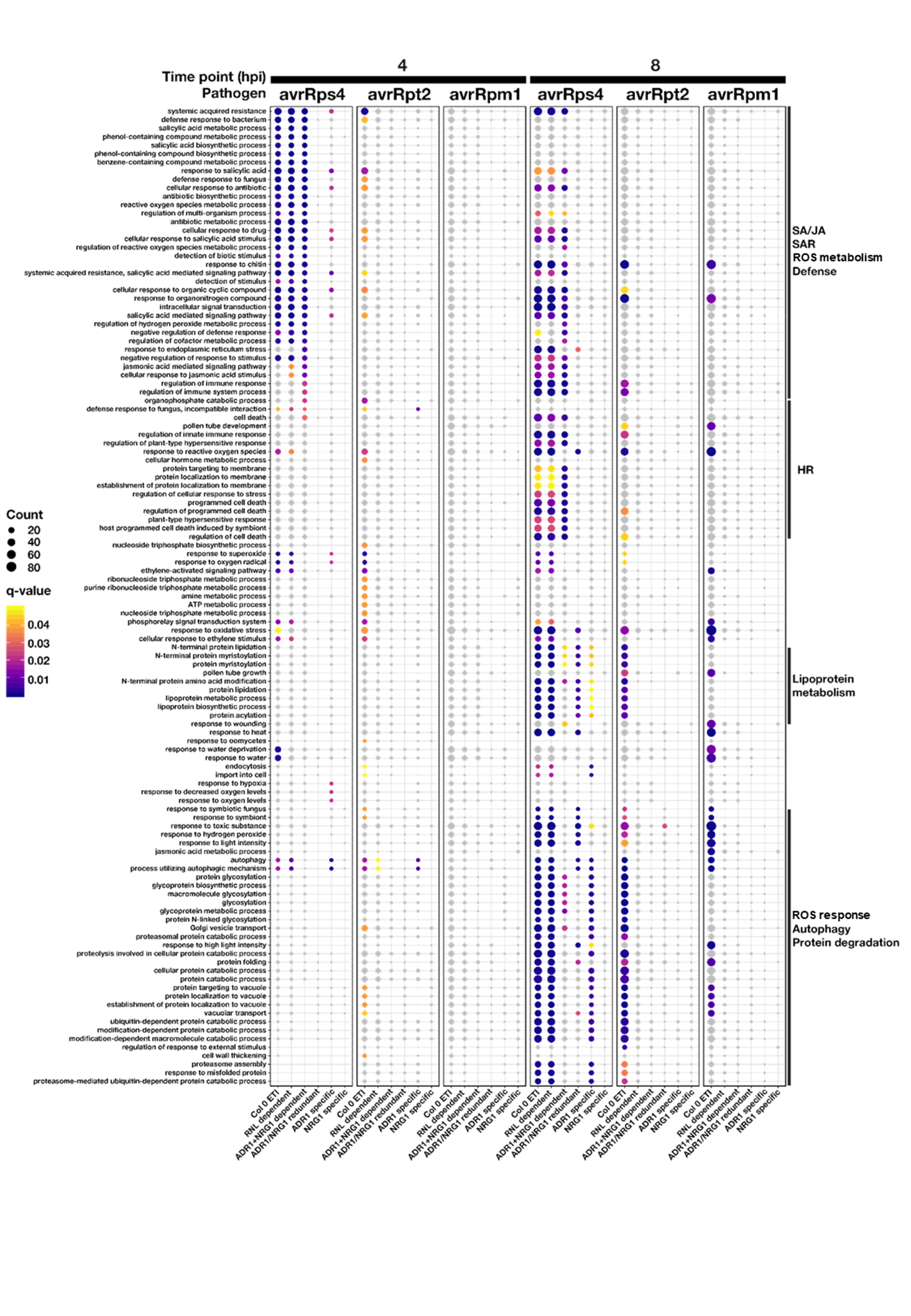

Supplement: S4 Fig — GO categorization of ETI-up-regulated genes. “Col-0 ETI” refers to the “ETI-regulated” gene set of Col-0. Activation of RPS4/RRS1, RPS2, and RPM1 induce genes involved in the same processes. There is no category associated to “ADR1 specific” that is distinct from the ones regulated redundantly or synergistically by NRG1s and ADR1s. For details see S2 Dataset. Underlying numerical data are provided in S1 Data. (TIF) [file pbio.3000783.s004.tif]

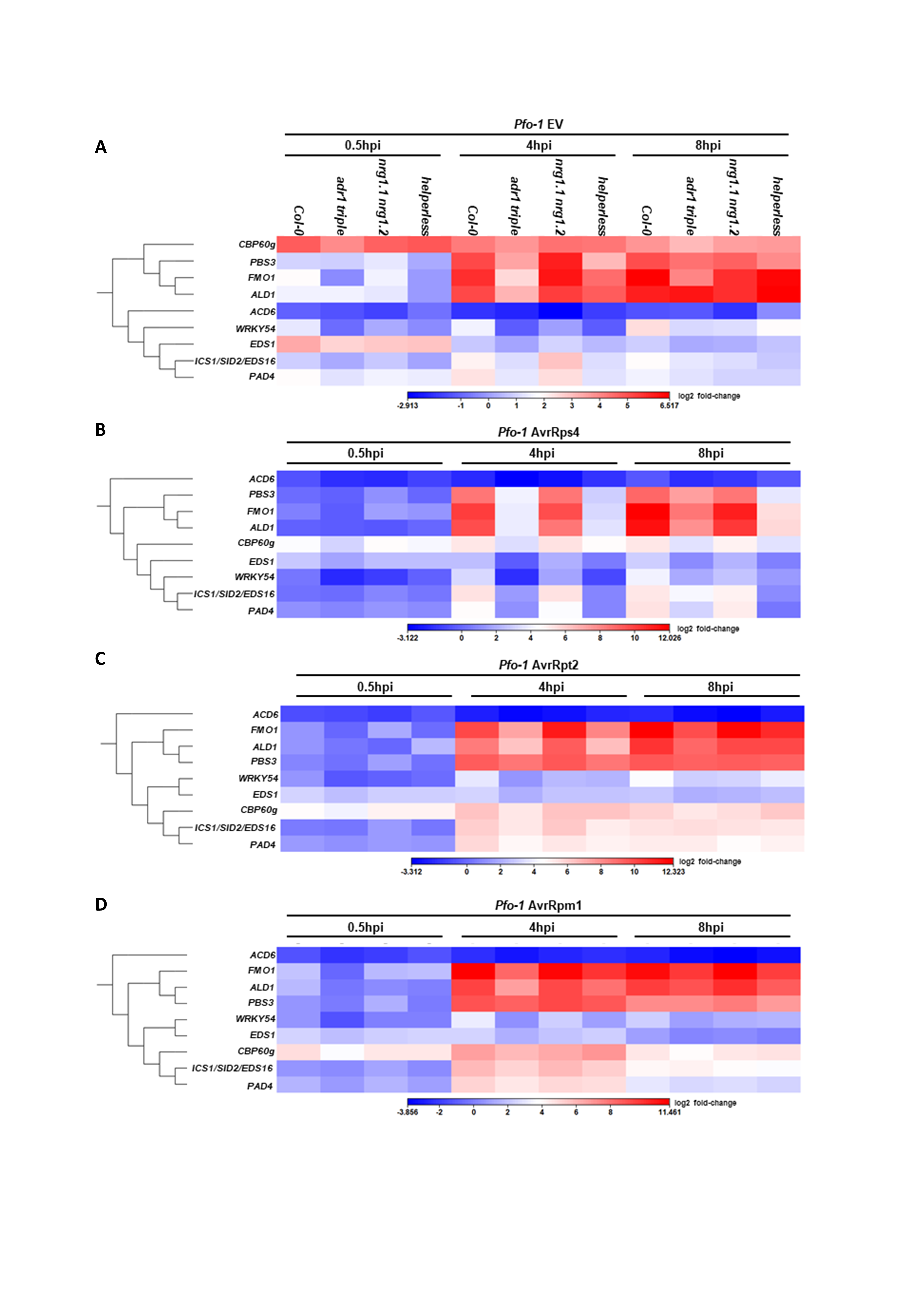

Supplement: S5 Fig — Comparison of SA-related gene expression in Pf0-1 EV (A), Pf0-1 AvrRps4 (B), Pf0-1 AvrRpt2 (C), and Pf0-1 AvrRpm1 (D) infiltrations at 0.5 hpi, 4 hpi, and 8 hpi. Genes are clustered according to their expression changes throughout the different samples and time points. Highest (red) and lowest (blue) log2 fold change is shown in heatmap. Visualization and clustering done with CLC Main workbench 20 (QIAGEN Aarhus A/S; www.qiagenbioinformatics.com). Underlying numerical data are provided in S1 Data. (TIF) [file pbio.3000783.s005.TIF]

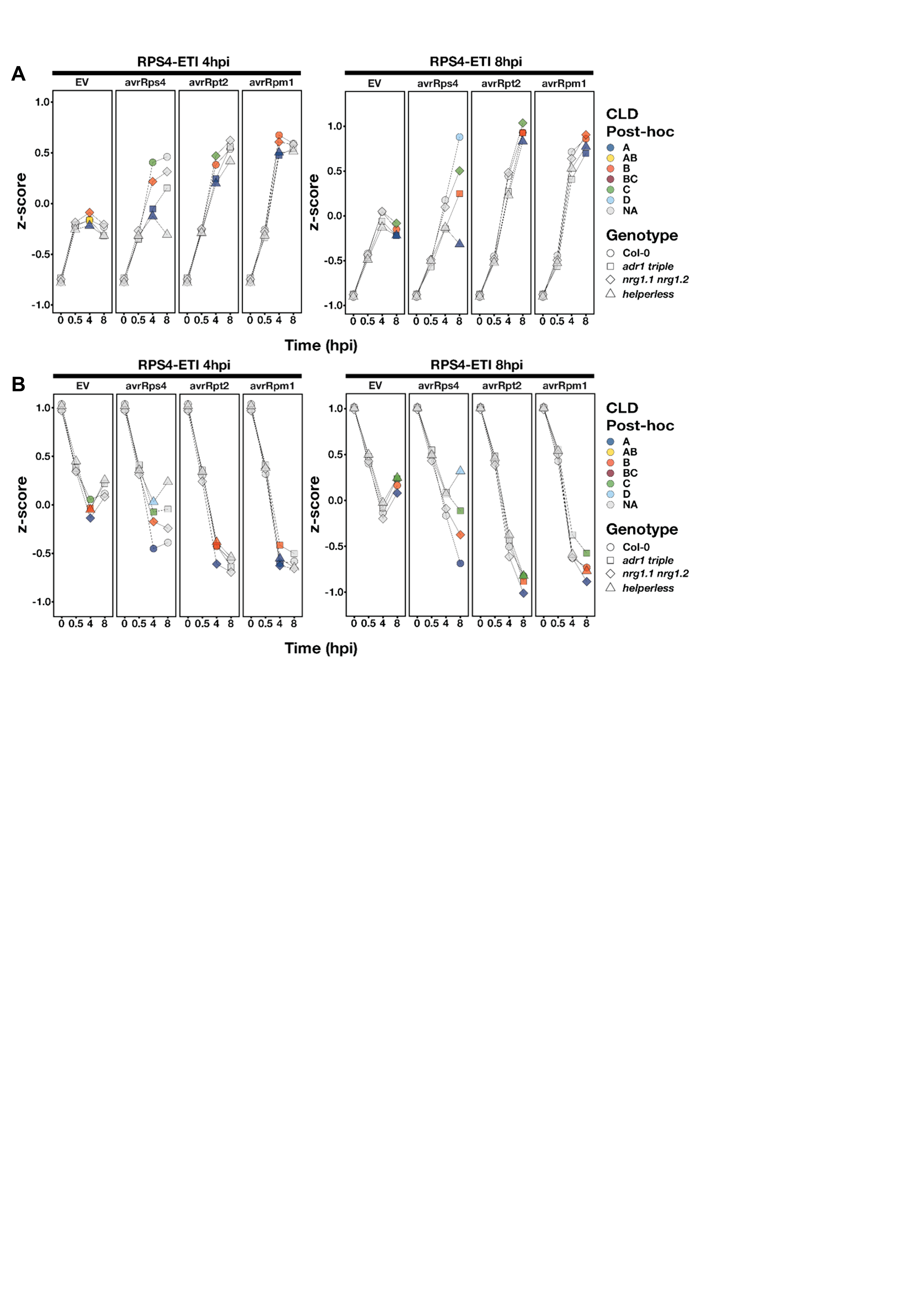

Supplement: S6 Fig — Normalized expression level of RPS4/RNLs up- (A) or down- (B) regulated genes at 4 and 8 hpi in Col-0 (circles), adr1 triple mutant (squares), nrg1.1 nrg1.2 (diamonds), and helperless (triangles) mutants, during Pf0-1 EV, Pf0-1 AvrRps4, Pf0-1 AvrRpt2, or Pf0-1 AvrRpm1 infection. Colors indicate a statistical difference (post hoc ANOVA, adjusted p < 0.05). Notably, RNL loss-of-function mutants affect gene expression quantitatively. This effect is most striking at 8 hpi during RPS4-mediated ETI but is also visible during RPS2- and RPM1-mediated ETI. Underlying numerical data are provided in S1 Data. (TIF) [file pbio.3000783.s006.TIF]
