# Supplementary material for: Two unequally redundant "helper" immune receptor families mediate Arabidopsis thaliana intracellular "sensor" immune receptor functions
Source: PLoS Biol. 2020 Sep 14;18(9):e3000783. doi: 10.1371/journal.pbio.3000783 (PMC7514072; doi:10.1371/journal.pbio.3000783)
Supplement: S1 Table — (DOCX) [file pbio.3000783.s007.docx]

**Supplementary Table S1: Expected expression profiles of ETI-induced genes according to their RNL-requirement.**

|  | \|  \| \| --- \| | **ETI-induced gene expression** | | | |
| --- | --- | --- | --- | --- | --- | --- |
| Category  Genotype | | Col-0 | *helperless* | *adr1 triple* | *nrg1.1 nrg1.2* |
| RNL independent | | **+** | **+** | **+** | **+** |
| RNL  dependent | ADR1/NRG1 dependent | **+** | **-** | **-** | **-** |
|  | ADR1/NRG1 redundant | **+** | **-** | **+** | **+** |
|  | NRG1 specific | **+** | **-** | **+** | **-** |
|  | ADR1 specific | **+** | **-** | **-** | **+** |

‘+’ and ‘-‘ indicate induced and uninduced expression respectively
