# Supplementary material for: Two unequally redundant "helper" immune receptor families mediate Arabidopsis thaliana intracellular "sensor" immune receptor functions
Source: PLoS Biol. 2020 Sep 14;18(9):e3000783. doi: 10.1371/journal.pbio.3000783 (PMC7514072; doi:10.1371/journal.pbio.3000783)
Supplement: S2 Table — (DOCX) [file pbio.3000783.s008.docx]

**Supplementary Table S2: Overview of helper RNL requirements in (auto-) immunity:**

**Response to pathogen infection:**

| pathogen | effector | *S*ensor NLR | *adr1 triple* | *nrg1 double* | *helperless* |
| --- | --- | --- | --- | --- | --- |
| *Pst* DC3000 | *AvrRpm1* | RPM1 (CNL) | **Bonardi et al. (2011):**  RPM1-mediated HR and resistance not compromised  **In this study:**  RPM1-mediated HR and resistance not compromised | **Castel et al. (2019):**  RPM1-mediated HR macroscopically not compromised, but compromised in conductivity measurements at early time points not at late time points  (*Pf0-1*)  **In this study:**  RPM1-mediated HR (*Pst* DC3000 and *Pf0-1*) and resistance (*Pst* DC3000) not compromised | **In this study:**  RPM1-mediated HR and resistance not compromised |
| *Pst* DC3000 | *AvrRpt2* | RPS2 (CNL) | **Bonardi et al. (2011):**  RPS2-mediated HR and resistance compromised  **Wu et al. (2019):**  RPS2-mediated resistance compromised  **In this study:**  RPS2-mediated HR compromised at early time points (10 hpi), not at late time points (24 hpi). RPS2-mediated resistance compromised | **Wu et al. (2019):**  RPS2-mediated resistance not compromised  **Castel et al. (2019):**  RPS2-mediated HR macroscopically not compromised, but partially reduced in conductivity measurements (Pfo-1).  RPS2-mediated resistance not compromised  **In this study:**  RPS2-mediated HR and resistance not compromised | **In this study:**  RPS2-mediated HR compromised at early time points (10 hpi), not at late time points (24 hpi). RPS2-mediated resistance compromised |
| *Pst* DC3000 | *HopZ1a* | ZAR1 (CNL) | **In this study:**  ZAR1-mediated HR and resistance not compromised | **In this study:**  ZAR1-mediated HR and resistance not compromised | **In this study:**  ZAR1-mediated HR and resistance not compromised |
| *Pf0-1* | *AvrPphB* | RPS5 (CNL) | **In this study:**  RPS5-mediated HR not compromised, but resistance compromised | **Castel et al. (2019):**  RPS5-mediated HR not compromised  **In this study:**  RPS5-mediated HR and resistance not compromised | **In this study:**  RPS5-mediated HR not compromised, but resistance compromised |
| *Pst* DC3000 | *AvrRps4* | RPS4 (TNL) | **Wu et al. (2019**):  RPS4-mediated resistance compromised  **Lapin et al. (2019):**  RPS4-mediated resistance compromised  RPS4-mediated HR not compromised.  **In this study:**  RPS4-mediated resistance compromised  RPS4-mediated HR not compromised | **Wu et al. (2019):**  RPS4-mediated resistance not compromised  **Castel et al. (2019):**  RPS4-mediated HR compromised (Pfo-1).  RPS4-mediated resistance not compromised (DC3000)  **Lapin et al. (2019):**  RPS4-mediated resistance not compromised.  RPS4-mediated HR compromised  **In this study:**  RPS4-mediated resistance not compromised.  RPS4-mediated HR compromised | **Wu et al. (2019):**  RPS4-mediated resistance severely compromised (stronger than adr1 triple)  **Lapin et al. (2019):**  RPS4-mediated resistance severely compromised.  RPS4-mediated HR compromised  **In this study:**  RPS4-mediated resistance severely compromised.  RPS4-mediated HR compromised |
| *Pst* DC3000 | *AvrHopA1* | RPS6 (TNL) | **Wu et al. (2019):**  RPS6-mediated resistance compromised | **Wu et al. (2019):**  RPS6-mediated resistance not compromised | **Wu et al. (2019):**  RPS6-mediated resistance compromised (stronger than in *adr1 triple*) |
| *Hpa* Emwa1 |  | RPP4 (TNL) | **Bonardi et al. (2011) and Wu et al.** (2019):  RPP4-mediated resistance compromised  **In this study:**  RPP4-mediated resistance compromised | **Wu et al. (2019)**  RPP4-mediated resistance not compromised  **Castel et al. (2019):**  RPP4-mediated resistance partially requires NRG1  **In this study:**  RPP4-mediated resistance not compromised | **In this study:**  RPP4-mediated resistance compromised |
| *Hpa Cala2* |  | RPP2 (TNL) | **Bonardi et al. (2011):**  RPP2-mediated resistance compromised  **In this study:**  RPP2-mediated resistance slightly compromised | **Castel et al. (2019):**  RPP2-mediated resistance partially requires NRG1  **In this study:**  RPP2-mediated resistance not compromised | **In this study:**  RPP2-mediated resistance severely compromised |
| *Albugo candida* |  | WRR4A^Col^ (TNL) | **In this study:**  WRR4A-mediated resistance not compromised | **Castel et al. (2019):**  NRG1s partially required for WRR4A-mediated resistance  **In this study:**  WRR4A-mediated resistance not compromised | **In this study:**  WRR4A-mediated resistance compromised |
| *Albugo candida* |  | WRR4B^Ws-2^ (TNL) |  | **Castel et al. (2019):**  NRG1s required for WRR4B-mediated resistance |  |
|  |  | CSA1 (TNL) |  | **Castel et al. (2019):**  NbNRG1 required for CSA1-induced HR |  |
|  |  | SOC3 (TNL) |  | **Castel et al. (2019):**  NbNRG1 required for SOC3-induced HR |  |
|  |  | CHS1 (TIR-NB) |  | Castel et al. (2019):  NbNRG1 required for CHS1-induced HR |  |
| *Pst* DC3000 | EV |  | **Bonardi et al. (2011)**  Enhanced susceptibility  **In this study:**  Enhanced susceptibility | **In this study:**  Resistance not compromised | **In this study:**  Enhanced susceptibility |
| *Pst* DC3000 cor- | lacks coronatine |  | **In this study:**  Enhanced susceptibility | **In this study:**  Resistance not compromised | **In this study:**  Severely enhanced susceptibility |
| *Pst* DC3000 Δ*hrcC* |  |  | **In this study:**  Resistance not compromised | **In this study:**  Resistance not compromised | **In this study:**  Resistance not compromised |
| *Hpa* Emco5 |  |  | **Bonardi et al. (2011)**  Enhanced susceptibility |  |  |
| *Psm* ES4326 |  |  | **Wu et al. (2019):**  Enhanced susceptibility | **Wu et al. (2019):**  Resistant | **Wu et al. (2019)**  Severely enhanced susceptibility |

**Autoimmunity:**

| Autoimmune mutant | *adr1 triple* | *nrg1 double* | *helperless* |
| --- | --- | --- | --- |
| *snc1* | **Dong et al. (2016)**  ADR1s largely required | **Wu et al. (2019)**  NRG1s partially required | **Wu et al. (2019):**  *snc1* auto-immunity fully suppressed |
| *chs2-1* | **Dong et al. (2016)**  ADR1s required: complete suppression |  |  |
| *chs3-1* | **Dong et al. (2016)**  Weak or partial suppression |  |  |
| *slh1-9* | **Dong et al. (2016)**  ADR1s partially required |  |  |
| *uni-1D* | **Dong et al. (2016)**  Weak suppression |  |  |
| *chs3-2D* |  | **Wu et al. (2019)**  NRG1s completely required  **Castel et al. (2019):** NbNRG1 required for chs3-2D induced HR |  |
| Snc2-1D |  | **Wu et al. (2019)**  NRG1s not required |  |
| *mekk1-5* (SUMM2 (CNL)) |  | **Wu et al. (2019)**  NRG1s not required |  |
| *chs1-2* |  | **Wu et al. (2019)**  NRG1s not required |  |
| ADR1-L2-D484V |  | **Wu et al. (2019)**  NRG1s not required |  |
| CSA1 (TNL) |  | **Castel et al. (2019):**  NbNRG1 required for CSA1-induced HR |  |
| SOC3 (TNL) |  | **Castel et al. (2019):**  NbNRG1 required for SOC3-induced HR |  |
| CHS1 (TIR-NB) |  | **Castel et al. (2019):**  NbNRG1 required for CHS1-induced HR |  |
| CC MLA10 (CNL) |  | **Castel et al. (2019):**  NbNRG1 not required for MLA7 CC-induced HR |  |
| RPM1-D505V (CNL) |  | **Castel et al. (2019):**  NbNRG1 not required for RPM1-autoactivity |  |
| RPS5 (CNL) |  | NbNRG1 not required for cell death activity upon effector-dependent activation |  |
