# Supplementary material for: Two unequally redundant "helper" immune receptor families mediate Arabidopsis thaliana intracellular "sensor" immune receptor functions
Source: PLoS Biol. 2020 Sep 14;18(9):e3000783. doi: 10.1371/journal.pbio.3000783 (PMC7514072; doi:10.1371/journal.pbio.3000783)
Supplement: Attachment — Submitted filename: Response to Reviewers Saile and Jacob et al.docx [file pbio.3000783.s013.docx]

**Response to the Reviewer comments on Saile and Jacob et al.,**

**PLOS Biology Submission (PBIOLOGY-D-20-00510R1) - [EMID:695d16d112c710ff]**

--------------------------------------------------------------------------------------------------------------------------------------

**Reviewer #1:**

The manuscript by Saile, Jacob et al. deals with the functional characterization of helper NLRs belonging to the RNL class in Arabidopsis thaliana. There are two different families of RNLs in Arabidopsis, the ADR1s and NRG1s. The three ADR1s are dispersed in the genome, and triple mutant plants were previously analyzed in detail (e.g. Bonardi 2011). However, analysis of NRG1s was previously hampered by tight genetic linkage. With increased availability of tools for targeted mutagenesis, several groups generated nrg1 mutant lines in N. benthamiana (Qi et al. PNAS, Castel et al. NPH) and/or Arabidopsis (Wu et al. NPH, Castel et al. NPH, Lapin et al. TPC), and also lines lacking both NRG1s and ADR1s were generated (Wu et al, Lapin et al). Analysis of these mutant lines revealed that

i) NRG1 appears essential for all immune responses mediated by TNLs (but not CNLs) in N. benthamiana (Qi et al, Wu et al)

ii) NRG1s are also important for a number of TNL-mediated resistance responses in Arabidopsis (e.g. RPS4/RRS1, RPP1), but some TNLs also signal via ADR1s. Resistance mediated by CNLs is not (RPS5, MLA) or only slightly (RPM1, RPS2) impaired (Castel et al)

iii) Different TNLs show various degrees of dependence on either ADR1s or NRG1s (mainly tested with autoimmune outputs), and ADR1s and NRG1s appear to function independently or synergistically in "basal resistance" (Psm) or TNL-mediated (RPS4, RPS6) resistance (Wu et al)

iv) One intriguing finding was that, in RPS4/RRS1-mediated resistance, ADR1s are more important to limit bacterial growth than NRG1s, but cell death was strongly reduced in nrg1 mutant, but not adr1 mutant plants (Castel et al, Lapin et al). This observation sparked the hypothesis that NRG1s are required for cell death and ADR1s for resistance signaling, implicating also that these are two independent processes.

The current manuscript provides a systematic and detailed comparison of Arabidopsis adr1 and nrg1 mutant lines with a "helperless" quintuple mutant line. In a first section of the manuscript, mutant lines are used in infection and HR assays to probe the requirement of a number of immune receptors for the helpers. Presented data confirms and extends previously reported findings, and reveals important contributions of NRG1s to several immune responses that only become visible in absence of ADR1s in the helperless line. In contrast to specific functions of NRG1s in cell death and ADR1s in resistance proposed before, the authors favor a model of genetic redundancy and synergism, with a certain degree of specialization visible in some comparisons.

In a second section of the manuscript, different mutant lines are used for rather large scale transcriptomics (312 samples?).

We indeed had 312 samples in total for our large scale transcriptomic analysis: 4 genotypes (Col-0, adr1 triple, nrg1 double, helperless) were tested with 4 infections (EV, avrRps4, avrRpt2, avrRpm1) and samples were taken at 3 different timepoints (0.5, 4 and 8hpi), with 2 samples for each experiment and this was repeated 3 times (4x4x3x2x3 = 288) and for time point 0 this was 4 genotypes x 2 samples x 3 repeats (4x2x3=24) makes in total 312.

This section globally supports findings of immunity assays etc, and further corroborates a minor contribution of hNLRs also to CNL-mediated resistance (RPM1, RPS2). An important feature of the underlying experiments is that modified Pseudomonas fluorescens strains were used - reducing the variables indeed to the one effector that is translocated (or not) by the bacteria in individual treatments. This section provides important information and conclusions, e.g. a lacking specialization of NRG1 or ADR1 regulons (arguing against functional specialization of the hNLRs) or overall resemblance of the hNLR-regulated transcriptome to a "classical" CNL transcriptome. It is my impression that this 2nd section of the manuscript requires substantial revision, in which the authors should improve the structure and also apply reductionism to guide readers towards the essential. I also feel that explanations in the main text, figure legends and methods for this section require improvement, but must admit that I am myself not a specialist for transcriptomics - other reviewer(s) may be more helpful here.

Overall, the manuscript may be considered as lacking novelty, as large parts confirm recent reports on helper functions. At the same time, the fine-grained transcriptome analyses adds to previous data, and the authors provide an alternative hypothesis on functions of hNLRs - not specialized for resistance (ADRs) and cell death (NRGs), but rather redundant and/or synergistic, with some sensor NLRs preferentially using one or the other helper class. It is important to actually provide this alternative hypothesis, as whether or not HR and resistance belong to the same "program" or not is a notorious subject. Thus, it definitively adds new aspects.

We value the very constructive feedback and comments made by this reviewer and appreciate the positive perception of our manuscript. We carefully read all the comments/suggestions and discussed them between all authors. We did include/incorporate them to make required changes. We made substantial modifications to the text to improve the structure and present the data in a more funneled way. We deleted parts of the text that were not essential to the comprehension of the study and simplified, edited or deleted figure(s)/tables (e.g. simplification and modification of figure S5, deletion of main figure 5). We added a table (table 1) and supplementary dataset (S5) to present the transcriptome data in a more efficient and summarized manner and also included two more experiments in the ‘infection’ part of the manuscript (Figure 3C - DC3000 *hrcC*- infection- and 3D and E - *Alternaria brassicicola* infection) that we think further supports our conclusion of the function of helper NLRs during (basal) immunity.

*Major points:*

-	The 2nd section of the manuscript is extremely difficult to read and would benefit from more reflected sub-structuring, as a (crude, un-reflected) suggestion i) presentation of the transcriptomics study in general, ii) hNLRs and PTI, iii) hNLRs and ETI, iv) specialization etc

We appreciate the suggestion and we changed the structure of this section and modified the text to make this section easier to read.

-	Supplemental figures are not cited in order (why?).

We thank the reviewer for this comment. We have changed the order of the appearance of the supplementary figures to fix this issue.

-	Supplemental figure S2 is not referred to at all

Thank you for highlighting this point. We have included a short paragraph about RNL function during PTI in the results part of the manuscript in which we refer to this figure.

-	The simple amount of supplemental data appears overwhelming, and a large portion of the results presents mainly data included in supplemental figures.

We value this comment by the reviewer, but we do think that the amount of data, although presented in supplementary figures, is required for readers to understand our logic and to be fully able to follow our interpretations. Further, there is only one supplementary figure for each main figure and thus, we do not think that this amount of (supplemental) data is too much. Additionally, we have modified figure S5 to present the included data in a more streamlined way and substituted figure S3 by the supplementary table S4.

-	Supplemental figure S3 is used to illustrate expression profiles underlying categorization for further analyses. However the graphs shown in this figure are composed of made-up data entered to Excel (if I get things right), the y-axes do not have a unit, and there are even error bars (derived from?). This suggests that "true" data is at the basis of the graphs, which is inappropriate. The same message could be illustrated in form of a table or a (flow) scheme.

We apologize for the confusion this supplemental figure S3 may have caused. There is no actual data on the basis of these graphs, this is right. We agree with the reviewer that there are other ways of illustrating the expected expression values and therefore, we have deleted figure S3 and substituted it by a table (supplementary table S4).

-	line 304: what is meant with Table 1 here? Not that simple/obvious to come from the Venn diagrams to the percentages mentioned in the same sentence.

Thank you for this important note. Table 1 was not submitted by mistake in the first submission. We have included it in the revision. Table 1 shows the fractions of ADR1 and/or NRG1 synergistic, redundant and specific gene upregulation during ETI. The table displays (i) the total number of genes being specifically upregulated during a certain ETI response which can already be seen in the Venn diagram, (ii) the number of genes being upregulated during a certain ETI response in an hNLR-dependent manner which can also be found in the Venn diagram and (iii) the calculated percentages which are not included in the Venn diagrams. On presentation of table 1 we assume that it will be possible to understand the percentages mentioned in the text.

-	~ line 307: "16.8 % of the response was lost in helperless (5E, 5F). It is the same type of info as previously (lines 302-304, where figure 4D was cited), but now figures 5E,F are cited? And again Table 1?

Thank you for making this note. Figure 5 was more a graphical/illustrative view of the data. We have deleted figure 5 and refer in both cases to figure 4D and table 1.

There are also 873 genes that are upregulated (in this comparison) in helperless, but not Col. Which is even more than those genes upregulated in Col, but not in helperless. Any comment on that?

We thank the reviewer for this comment. We have also noticed this. However, it is at this time not known whether these genes reflect the action of the bacterial effector or results from the overcompensation of the loss of RNLs. This remains to be elucidated, but is not a focus of this manuscript. However, we added the following part to the manuscript to address this concern:

“Many genes were found differentially expressed specifically in the *helperless* mutant (Fig 4D). “

-	line 333 and following: Again, what is Table 1?

Table 1 was not submitted by mistake in the first submission. We have included it in the new file.

-	Figure 5: The bar graphs only represent the numbers directly printed above. At the same time, the text only cites percentages, which can be calculated from these numbers. This is confucing, and I do not see any point for displaying these data as graphs. A table could display these data more efficiently, and would give the simple opportunity to both indicate absolute numbers and percentages - which are mentioned in the text (lines 333 and following). I propose to convert data into a table to eliminate Figure 5 (again, y-axes go without units). Also, the entire paragraph about RNL-specific regulons is lengthy

We thank the reviewer for this important comment and have made specific changes to the manuscript. The table 1, which was omitted by mistake in the first submission fulfills the very purpose of summarizing the data. We added the table to present all the data in an efficient way and eliminated figure 5 as suggested. We modified the text of the RNL regulon section to shorten it and thus hope to made it clearer.

-	lines 378 and following: "RNLs regulate the same genes as CNLs (S4 Fig)". Looking at the figure S4 and also considering colors (q value), this is not obvious. It is visible that the counts (the size of the circles) are similar… but still. The legend (methods) should include some comment on q-value, and I am wondering whether it might not be possible to display this type of data in a different (simpler) manner to make the conclusion more obvious to extract, more accessible.

Thank you for this note. We agree that this is difficult to extract from this figure. This figure only aims at presenting the different GO terms and processes associated with the ETI regulated genes and the RNLs regulons. This figure is only used for descriptive purposes and not to claim that RNLs regulate the same genes as CNLs. It is important to understand what motivated the other analyses that do show that RNLs regulate mostly the same process than CNLs. We added a dataset (Suppl dataset S5) to show the details of the gene ontology analysis and modified the text to make it clearer that we do not use this data to make this claim.

-	Figure 6, legend: (A,B) is correct, (B,D) should say (C,D)

Thank you for bringing this up. We have changed the typo accordingly.

-	One important conclusion of the transcriptome analysis is that expression changes mediated by RNLs (and thus by TNLs as sensors, in the case of RPS4/RRS1) are similar to those of classical CNLs. Reports on structural elucidation of the resistosome from 2019 (Wang et al, Science) put these findings to some extent into context, as further reports suggest that the resistosome mechanism might be one major mechanism for signaling by different CNLs (eg Hadachi et al., eLife 2019; Li, Habring et al. Cell Host Microbe 2020). These reports should be discussed and also be included in intro.

We thank the reviewer for this comment and suggestion. We have mentioned the oligomerization requirement for NLR signalling in the introduction now and cited the relevant papers. We also further discuss the findings of the ‘resistosome’ papers in context of our results in the discussion part.

-	Similarly, a recent report on a Pst effector compendium (Psy-Tec; Laflamme et al. Science 2020) showed an interesting positive correlation between the strength of resistance and the occurrence of macroscopic HR - further corroborating the notion that these are not distinct pathways as concluded from the transcriptomics. This should also be included in the discussion.

Thanks for this comment. We now have included this very interesting new paper in our introduction and discussion where we talk about basal resistance and PTI.

-	line 464: the qi 2018 PNAS reference should also be listed here, as NRG1 was not analyzed in the mentioned reference

Thanks for bringing this up. We have added the specific reference accordingly.

-	line 522 and following: It is stated in the article that "basal resistance" also includes weak ETI (lines 209-10). Thus, the statement made here is contradictory. This is actually taken up again in line 537, but now with a different statement.

We value the reviewer’s point made here. It is true that basal defence is a combination of PTI minus ETS plus remaining weak ETI (as also supported by the data presented in Laflamme et al., 2020 Science). Thus, basal defence is most likely initiated by the combined action and signalling of cell surface-localized RKs and RLPs and weak intracellular NLRs. Our data and published results (e.g. Bonardi et al., 2011 PNAS) suggest that RNLs are not required for RK signalling. However, whether this holds true for all RKs and for RLPs awaits further examination. Most likely the Pseudomonas-Arabidopsis pathosystem is not the best choice to analyse this, since resistance against virulent Pseudomonas relies on RK function (Albert et al. Plant Physiol. 2019;182:1582-1596 – Figure 2). This is further supported by our now included Pst DC3000 *hrcC* data. We rephrased the specific sections in the text.

-	line 551 and following: Nicotiana benthamiana lines are mentioned in the methods, but were not used in experiments, if I am not mistaken

Thank you for this comment. We deleted this section in the method part of the manuscript.

*minor points:*

-	line 58 "…to dampen PTI [5]". This may not be the most appropriate citation here, as the cited review focusses rather on effector functions different from PTI suppression.

Thank you for bringing this up. We have changed the reference accordingly.

-	lines 64 - 65: Consider re-phrasing. The sentence may imply that there are 3 classes of NLRs, and these are further divided in sub-classes.

Thanks for bringing this up. We modified the sentence accordingly to better reflect the division of plant NLRs into two major types (CNLs and TNLs) and to point out that the RNLs are a unique subclade of the CNLs and do not represent a third class of plant NLRs.

-	lines 78 - 80: The statement about the used nomenclature is a bit confusing, as no alternative nomenclature is mentioned. It might be helpful to refer to the alternative NRG1A etc names used e.g. in Wu et al.

We thank the reviewer for this comment and have included a mentioning of the alternative nomenclature of *NRG1* genes in the text.

-	line 104: Consider rephrasing. It is rather the (immune) phenotype of helperless mutant plants that was examined than the helperless phenotype.

We appreciate this note and have changed the text specifically.

-	line 132: Shouldn't it say "different requirements for"?

Thanks for this note. We corrected the phrasing here.

-	line 138: cooperative function of the TNLs

We thank the reviewer for picking this up. We have changed ‘...function of the TNL pairs RPS4/RRS1.’ Into ‘...function of the TNLs RPS4 and RRS1.

-	lines 150 - 152: Results referred to in this phrase do not indicate recognition of AvrRps4 by another TNL (pair), as a direct comparison with DC3000 would be required for this statement. Also, experiments shown in Saucet 2015 (Nat Comms) suggest that this is not the case (equal susceptibility of rrs1a rrs1b to AvrRps4 and DC3000). This suggests rather that additional (weak) recognition events may be impaired in the helperless line

We thank the reviewer for bringing this up. We agree that without the direct comparison to the DC3000 EV control in the same experiment we cannot make such a claim. We changed the text in the result section accordingly.

-	line 154: [10, 12] Citation [10] is inappropriate for this statement, as an rrs1a rrs1b line was not used in this study. The Saucet 2015 reference should be used here.

We appreciate this critical and correct comment of the reviewer and have deleted reference [10] here and added the reference mentioned by the reviewer: Saucet et al., 2015 Nature Comm.

-	line 198: I think that journal policy requires figure panels to be referred to in order. Here, E is cited before D.

Thank you for bringing this up. We changed the figure array accordingly.

-	line 217 …helperless plants

Thank you for this note. We changed the typo accordingly.

-	line 232 and following: figure 3 (or rather data therein) cannot demonstrate anything in respect to Hpa, as only Pst was used in the experiments. The comment on a "slight contribution" is confusing, as no effect is stated a few lines before. Also, this is not supported by statistics and not convincing in the figure.

We thank the reviewer for bringing this up. We agree that based on the data presented in the old and new figure 3 we cannot make any claims with respect to defence against Hpa. We have changed the section accordingly and also rephrased the comment on “slight contribution” to now justify the presented data.

-	line 274: 4B cited before 4A

Thank you for bringing this up. We changed the figure array accordingly.

-	line 283: infectionsin

Thank you for this note. We corrected the typo.

-	lines 287 and following: The statement about the Pf0-EV transcriptome at later time points should be incorporated in the discussion

We thank the reviewer for this comment. However, we do want to make this statement here, since we are not discussing this again in the discussion part.

-	line 294 and following: this clustering is really difficult to see and extract from the PCA.

We appreciate this comment by the reviewer. We deleted the text accordingly to make it easier for the reader to understand and extract the point we want to communicate here.

-	line 310: "contribute weakly to basal resistance". Shouldn't it rather say PTI, when using Pfluorescens?

We thank the reviewer for bringing this point up. We indeed test for PTI responses when using the *Pf0-1 EV* system and we also mention that in the previous section (see second paragraph of section entitled ‘RNL requirement in transcriptional reprogramming during PTI’). We therefore made the mentioned change and rephrased this in the text accordingly.

-	ROS response should be spelled in capitals in S4

Thank you for picking this up. The figure was modified accordingly.

-	line 390: "the expression … could be efficiently induced/repressed". Rephrase.

Thanks for bringing this up. We changed the specific part accordingly.

-	line 604: infections

Thank you for this comment. We changed the typo accordingly.

figure legends:

-	Figure 1B: Statement about means not clear; consider rephrasing.

We value the reviewers comment and changed the figure and figure legends accordingly. In the updated figure we provide the means of Sp./cotyledon as squares ± SE and described it properly in the figure legend.

-	Figure 1C: "Activated NLRs are in parenthesis." is a bit confusing. Maybe add something like NLRs activated in infection experiments shown in A-C are indicated in parenthesis.

We agree on the reviewers’ suggestion and rephrased the sentence accordingly.

-	figure s1: the " x 3 ml" in the unit definition appears a bit odd

Thank you for making this point. We changed the units accordingly.

-	many figure legends could be more detailed

Thank you for this note. We included more details in the figure legends where necessary and hope that the provided information is sufficient.

**Reviewer #2:**

NLR proteins are central to plant immunity and consequently are of enormous academic and agricultural importance. The RNL subclass of NLR proteins are emerging as key components for the function of numerous NLRs. However, despite their importance, there is currently only limited information regarding RNL activities associated with plant immune function. This manuscript provides deep new insights into RNL function associated with different branches of plant immunity.

It is therefore both important and timely. The manuscript is both written and presented to a high standard. It is a beautiful paper full of important information that will be of great interest to the community.

Consequently, I would strongly recommend this paper is accepted post a minor revision.

The first link of the RNL class of proteins to plant disease resistance was by Grant et al. 2003.

It would therefore be helpful for readers if this paper was cited.

We thank this reviewer for the positive assessment of our manuscript. We appreciate the comment on the first link of the RNL class (ADR1 to be specific) to plant disease resistance and we added the Grant et al., 2003 citation to the introduction accordingly.

**Reviewer #3:**

The manuscript by Saile et.al reported an unequally redundant role of two helper NLR families ADR1s and NRG1s in Arabidopsis immune response. Plant NLR proteins functions as intracellular immune receptors that perceive pathogen effectors to induce ETI. Recently, two RPW8-like CC-NLR (RNL) families ADR1s and NRG1s were found serve as helper NLRs for sensor NLRs, especially TIR-NLRs (TNLs). However, the specificity and redundancy of these two helper NLRs remain less understood. By testing various pathogens and CNL-, TNL-typed effectors in adr1 triple, nrg1 double and helperless mutants, this manuscript reveals an unequally redundant role of ADR1s and NRG1s.

Although the major conclusion of this study that ADR1s and NRG1s are required downstream of all sensor TNLs to activate ETI, and that both RNL families are required to support the activation of some CNLs-initiated ETI, was established by Wu et al. and Lapin et al. before, the authors tested more different effectors to present specificity of ADR1s and NRG1s in different TNL and CNL signaling pathways. The time-resolved genome expression profiling data also give more information of helper NLRs' function in transcriptional reprogramming during basal resistance and TNL-, CNL-mediated immunity.

Minor:

No error bar and statistical analysis were shown in figure 1B and figure 2B. The authors also should indicate how many biological replicates were performed.

Thank you for making this point. Since figure 1B and 2B showed only the percentage of cotyledon/class, no error bars were shown. We changed the figure to include error bars on the means of spores/cotyledon. Means were also used for statistical analysis as indicated in the updated figure. Besides, we also added the number of biological replicates in the figure legend as suggested by the reviewer.

The eds1 mutants were tested in figure 1B, figure 2B and figure 3A. However, eds1 mutant results were not shown in figure 1A, figure 2A C D E, and figure 3B. It is important to compare all data equally.

We thank the reviewer for making this point. It is true that in figure 1B and 2B the *eds1-12* mutant was included as a susceptible control in addition to the respective NLR mutant (*rpp2* and *rpp4* in figure 1B and 2B, respectively). However, this control would not be necessary for our conclusions and is in addition to the necessary and sufficient (and included) NLR loss of function mutant controls. The *eds1-12* mutant is a fixed part of our Hpa infection analysis and included in all experiments, regardless of whether it is required or not, and therefore we decided to also include it in the figure panels presenting the Hpa infection experiments. In Figure 3 *eds1-12* is the only control for susceptibility and therefore required, as correctly mentioned by the reviewer. We changed the figure to include the *eds1-12* data that we did obtain while we performed the presented experiments. However, we did not collect day 0 data for all *eds1-12* replicates and therefore decided to leave the day 0 data out for all genotypes tested in order to make the figure more coherent.

The authors showed that helper NLRs ADR1s and NRG1s unequally and redundantly contribute for basal resistance. However, the helper NLRs do not function in flg22-induced ROS production. It seems further discussion is needed on how PTI is linked to helper NLRs**.**

We thank the reviewer for articulating this concern. We now show in addition to the flg22-induced ROS burst that RNLs are also not required for the growth restriction of *Pst* DC3000 *hrcC* (Figure 3C), a typical PTI response. These results suggest that RNLs do not significantly contribute to PTI. However, basal resistance, where we observe a function of the RNLs, is the outcome or sum of PTI minus ETS plus weak ETI and therefore, RNL function during basal resistance could be linked to a weak ETI activation, most likely mediated by unknown or (weak) NLRs (see e.g. Laflamme et al. 2020 or Thordal-Christensen 2020).

It is a little confused by the colors of arrows in figure 7. Are the pathways connected by the red arrow and the pathways connected by the black arrow are independent or integrated? The authors showed that the helper NLRs are involved in some of the CNL-mediated immunity. Why there is no arrow indicating connection between helper NLRs and CNLs?

Thank you for bringing this point up. We have tried to consider the issues brought up by this reviewer and hope to now have improved the readability of the working model (figure 7). The red arrows represent ETI specific signaling. PTI and ETI are integrated in this proposed model as was proposed before us. There is no arrow connecting RNLs to CNLs directly on purpose. We observed that RNLs are involved in full resistance in some plant-pathogen interactions involving also CNLs. RNLs are not strictly required for any CNL function, but they (seem to) behave as canonical CNLs. Therefore, RNLs and CNLs can be considered redundant and RNLs should be considered to act in parallel with CNLs and not downstream (or upstream). Thus, in the absence of data showing a direct link a model not linking CNLs to RNLs is representing the available data more realistically. However, in our model, RNLs are still indirectly linked to CNLs signaling as at least ADR1-L1 and ADR1-L2 act downstream SA, which is also a feature of CNL signaling, in particular RPS2. We modified the figure legend to try to reflect this better.

**Reviewer #4:**

In this manuscript, the authors further characterize the role of ADR1s and NRG1s in TNL- and CNL-mediated ETI responses and also in basal resistance.

In agreement with the previously published report (Ref #10), the data presented in this paper also show that ADR1s and NRG1s function in an unequally redundant manner in RPS4/RRS1- and RPP2-mediated resistance. Furthermore, the data presented indicate that ADR1s and NRG1s function redundantly in TNL WRR4-meidated resistance.

Consistent with the previous report (Ref #10), NRG1s are required for RPS4-mediated HR and ADR1s are required for RPS2-mediated HR and disease resistance. In addition, the data presented also shows that ADR1s play a role in basal defense. Here, authors use Pst DC3000 cor- strain to uncover the role of NRG1 in basal defense.

The data presented clearly shows that ADR1s and NRG1s have no role in RPM1-, RPS5- and ZAR1-mediated ETI responses.

Most importantly in this paper, the authors present a significant amount of time-resolved transcriptome data to determine the role of RNL in shaping the transcriptome reprogramming during PTI and ETI. The data presented clearly shows a major requirement of RNL towards TNL-triggered transcriptome reprogramming and partial requirement in CNL-triggered transcriptome reprogramming.

We would like to thank this reviewer for the careful reading of our manuscript and the valuable input and comments.

However, RNLs play a limited role in transcriptional reprogramming during basal resistance. This is somewhat surprising given ADR1s and NRG1s role in basal resistance (Fig. 3).

We appreciate this idea. We and others observed that RNLs play a role in basal resistance to virulent pathogens (Wu et al., 2019, Lapin et al., 2019). However, in our transcriptome experiment we did not test for transcriptional reprogramming during basal resistance, since *Pfo-1* EV is not a virulent pathogen and as such is mostly comparable to the T3SS mutant *Pst* DC3000*hrcC-*. Infections with these pathogens induce a strong PTI response and therefore do not, as such, reflect what is happening during basal resistance, which is PTI minus ETS influenced by potential weak ETI. To confirm that RNLs are not involved in PAMP-triggered immunity and PTI-dependent transcriptional reprogramming, we tested resistance of the RNL mutants to *Pst* DC3000*hrcC*- (data presented in new Figure 3C). We found that RNLs were not required for resistance to *Pst* DC3000*hrcC-*. We also show that typical PTI signaling, e.g. from the RLK FLS2, does not require RNLs. We conclude that RNL involvement in basal defense is related to weak remnant ETI signaling triggered by *Pst* DC3000 effectors. This is consistent with the recent publication of Laflamme et al. (Laflamme et al., 2020 Science) showing that among *Pst* DC3000 effectors, HopB1 is recognized by the TNL BAR1/At5g18360 and can contribute to resistance to *Pst* DC3000 although BAR1 does not trigger HR. The same seems to be true for other Pseudomonas pathogens of which more than 90% have one or more effectors belonging to an effector clade that is potentially recognized by one of the NLRs described (and identified) in Laflamme et al., 2020. We added a new panel (panel C) in fig3 and the result and discussion section were modified accordingly to reflect this model.

Text and conclusions are difficult follow with respect to transcriptome data.

We appreciate this comment and tried to simplify the text and conclusions where we thought it was necessary and possible. We hope that the text and our conclusions are easier to read and understand now.

Most of the data shown in figure is based on number of genes that are differentially regulated in wild type and different mutants. Some details on what kinds of genes are there in these gene sets will help readers to appreciate the data. Supplementary Table S2 and S3 fails to open when downloaded; it says unsupported format.

We apologize for the issue with the supplementary table S2 and S3, also we do not know what went wrong here. All the other reviewers did not mention any problems in downloading, opening and viewing these tables and nor did we when trying this from the PLOS server. The details of the genes that were found in the different ‘gene sets’ can be found in these tables and also in Supplementary data sets, and thus we do think it is not necessary to mention details of certain genes of a certain gene set specifically. All this information can be extracted from the data. In our manuscript we would like to focus the attention on the (more general) finding that RNLs act as canonical CNLs during TNL-mediated ETI, at least in the transcriptional reprogramming, we do not want to focus, in this manuscript, on specific genes or pathways that could be e.g. only RNL dependently regulated.

S2 Figure data is not presented in the text.

Thank you for highlighting this point. We included a paragraph about RNL function during PTI in the manuscript in which we refer to this figure.
