# Supplementary material for: Two unequally redundant "helper" immune receptor families mediate Arabidopsis thaliana intracellular "sensor" immune receptor functions
Source: PLoS Biol. 2020 Sep 14;18(9):e3000783. doi: 10.1371/journal.pbio.3000783 (PMC7514072; doi:10.1371/journal.pbio.3000783)
Supplement: Attachment — Submitted filename: Response to reviewers Saile and Jacob et al revision_4.docx [file pbio.3000783.s014.docx]

**Response to the editorial comments on Saile and Jacob et al.,**

**PLOS Biology Submission (PBIOLOGY-D-20-00510R3) [EMID:6ba6ed8a32580b99]**

--------------------------------------------------------------------------------------------------------------------------------------

**Editorial remarks:**

We would appreciate your prompt cooperation with our requests at your earliest convenience. If you have any questions or require clarification, please do not hesitate to get in touch.

1. Please ensure that all main figure files follow PLOS naming conventions in the File Inventory. The file name should be in the format “Fig1.tif” and the file description should be “Fig 1”.

We think that we have fulfilled the naming conventions for all our main figures accordingly. Figure files are named “Fig1.tif” and the figure caption starts with e.g. “Fig 1:”. We are not sure what is meant by file description and therefore were not able to fulfil this request.

2. Please note that your figures do not currently comply with our publication requirements. For further instructions to assist with your Figure revisions, please refer to our Figure Guidelines at: http://journals.plos.org/plosbiology/s/figures.

To comply with the PLOS publication requirements for main figures we have used the online tool PACE as suggested by the PLOS Biology homepage. We have adjusted all main figures in regard to size, resolution, 2-point withespace border and RGB colour mode.

If you need assistance, please contact us directly at graphics@jjeditorial.com so we can further help with this matter. Please send any relevant source files and tell us what application(s) and what operating system you are using.

- Please crop Figures 1, 2, 3, 5, and 6 to remove the whitespace around the figures themselves. PLOS recommends a 2-point whitespace border, but more will cause the figures to be incorrectly placed in the published PDF.

See response to point 2.

- Panels A and B in Figure 1, panels A-C in Figure 2, and panels A-D in Figure 3 appear pixelated/low quality. Please remake these parts of the figures using the original source, using the PLOS figure guidelines to ensure quality is preserved [http://journals.plos.org/plosbiology/s/figures].

See response to point 2.

- Figures 4, 5, and 6 exceed the dimension limits for figures. Figures must be less than 19.05 cm in width and less than 22.225 cm in height (excluding white space around your artwork). Please make the necessary adjustments and re-upload your figure(s).

-In Photoshop: Image→Image Size. Make sure the Resample Image checkbox is NOT checked. If the Width is over 19.05 cm, type 19.05 in the Width box (19.05 cm is our maximum allowable width for figures).

See response to point 2.

-In GIMP: Image→Scale Image. Set the units of measurement, in the pull down menu next to Height, to millimeters. If the Width is over 190.5 mm, type 190.5 in the Width box (19.05 cm is our maximum allowable width for figures) and hit Tab. The new Height of the figure will appear, scaled proportionately to the change in Width.

- Figures 4, 5, and 6 look like they were originally at a lower resolution and will appear blurry when published. This error cannot be fixed by simply increasing the resolution of your figures with image editing software. Instead, please carefully review the PLOS Figure Guidelines and provide figure files that were originally created at a minimum of 300 pixels/inch (ppi) and that are sized between 6.68-19.05 cm wide and no more than 22.225 cm tall (excluding any white space around your artwork). Additionally, please ensure your figures are in RGB colour mode.

See response to point 2.

3. Please ensure that all supporting information legend titles use the following format verbatim in all in-text citations (including spelling and capitalization): S1 Fig, S1 Table, S1 Text, S1 Data, S1A Fig etc. Each file type should be numbered starting at 1.

We have made the requested changes in the main text and supplementary captions.

4. Please note that your submission cannot contain any references to generic "supporting information" or “supplementary” files and instead must refer to specific files using our naming convention (S1 Fig, S1 Table, S1 Text, S1 Data, etc.)

We have made the requested changes in the caption of supplementary data file S1 Data.
